# Delayed onset urticaria and symptomatic dermographism following COVID‐19 booster vaccination: A case series

**DOI:** 10.1002/clt2.12206

**Published:** 2022-11-12

**Authors:** Jakob Lillemoen Drivenes, Aleena Banerji, Anette Bygum

**Affiliations:** ^1^ Vestfold Hospital Trust Tønsberg Norway; ^2^ Division of Rheumatology, Allergy, and Immunology Massachusetts General Hospital Harvard Medical School Boston Massachusetts USA; ^3^ Department of Clinical Genetics Odense University Hospital Odense Denmark; ^4^ Clinical Institute University of Southern Denmark Odense Denmark

To the Editor

To date, more than 3.6 million persons in Denmark have received booster doses of COVID‐vaccine, of which the majority have been the Pfizer‐BioNTech's “Comirnaty” (86.5%) and Moderna's “Spikevax” (13.5%) vaccines.^1^ Cutaneous adverse events (CAE) following vaccination with the first two doses of COVID‐vaccine have been increasingly reported, but lately also CAE following booster vaccination has been observed.^2^, ^3^, ^4^, ^5^ In private dermatology practice, we have noticed an increasing number of patients with delayed onset urticaria and symptomatic dermographism following booster vaccination with Pfizer‐BioNTech and Moderna.

This case series is based on 16 consecutive patients with delayed onset urticaria after vaccination cared for in a single private dermatology practice with a catchment area of 90,000 patients. Data was prospectively collected in a 5‐month period from February to June 2022. Clinical and demographic data are presented in Table 1. The patient population was represented by 10 women and six men ranging in age from 20 to 73 years with a median age of 33 years. Thirteen patients had no previous medical history of urticaria and developed inducible urticaria and/or spontaneous urticaria post vaccination. The remaining three patients developed an exacerbation of their preexisting urticaria with newly developed symptomatic dermographism in two. The dermographism was evaluated by FricTest with four pins (Fric 4.0). One patient (number 16) later developed cutaneous lymphocytic vasculitis. All patients developed symptoms within 1 day to 3 weeks following booster vaccination with a median time of 14 days. Most patients had severe symptoms with six requiring acute doctor visits and four patients being admitted to the emergency room. All patients were treated with high‐dose antihistamines, and four patients received treatment with systemic corticosteroids. A total of five patients received further treatment with omalizumab. In all cases, the most likely provoking factor of the chronic spontaneous urticaria (CSU) and marked symptomatic dermographism was believed to be the COVID‐vaccine. No other plausible causes were found, despite a thorough medical history and routine blood tests following the international EAACI/GA^2^LEN/EDF/WAO guideline, and in many patients also more extensive blood testing and clinical workup. At follow‐up in August 2022, a total of four patients had experienced resolution of urticaria and dermographism after a median time of three months, while four patients were in clinical remission with antihistamines and omalizumab. Seven patients were still symptomatic despite therapy with antihistamines and omalizumab for symptom control. One patient was lost to follow‐up.

**TABLE 1:** Clinical demographic data. Ten patients (62.5%) were females, and six patients (37.5%) were males. Two patients (12.5%) had COVID‐19 prior to development of delayed urticaria/dermographism. Fifteen patients (94%) developed urticaria after the third dose of COVID‐vaccine and one patient (6.2%) developed urticaria after the second dose. Twelve patients (75%) received Moderna, and four patients (25%) received Pfizer‐BioNTech vaccine. Seven patients (43.7%) still experience symptoms from their delayed urticaria/symptomatic dermographism. 4 (25%) patients are in clinical remission with ongoing antihistamines and Omalizumab therapy. Seven patients (43.7%) had a history of an atopic disease.

Patient (Age/Sex)	History of COVID‐19	History of urticaria	Onset after vaccination	Delayed urticaria	Dermographism evaluated by FricTest	Vaccine dose	Vaccine	Treatment	Duration	Medical history
1 (31/M)	Negative	Negative	21 days	Negative	+	3^rd^	Moderna	AH	Ongoing symptoms despite AH (7 months)	Healthy
2 (33/F)	Negative	Negative	10 days	Negative	+	3^rd^	Moderna	AH + CS Omalizumab	Remission, ongoing therapy AH + Omalizumab (3 months)	Penicillin allergy with anaphylaxis NSAID intolerance with urticaria
3 (34/F)	Negative	Negative	14 days	+	+	3^rd^	Moderna	AH	Resolution after 3 months	Atopic dermatitis Allergic rhinitis
4 (28/F)	Negative	Negative	14 days	+	Negative	3^rd^	Moderna	AHOmalizumab	Remission, ongoing therapy AH + Omalizumab (4 months)	Atopic dermatitis Allergic rhinitis
5 (22/F)	+	Negative	7 days	+	+	3^rd^	Moderna	AHOmalizumab	Remission, ongoing therapy AH + Omalizumab (2 months)	Allergic rhinitis
6 (57/F)	Negative	Negative	14 days	+	Negative	3^rd^	Pfizer‐BioNTech	AH	Ongoing symptoms despite AH (7 months)	Atopic dermatitis
7 (20/F)	Negative	Negative	21 days	+	+	2^nd^	Pfizer‐BioNTech	AH + CSOmalizumab	Ongoing symptoms despite AH + Omalizumab (7 months)	Atopic dermatitis
8 (43/F)	Negative	Negative	14 days	+	+	3^rd^	Moderna	AH + CS	Ongoing symptoms despite AH (7 months)	Healthy
9 (36/F)	+	Negative	14 days	+	+	3^rd^	Moderna	AH	Resolution after 2 months	Rosacea
10 (33/M)	Negative	Negative	14 days	+	+	3^rd^	Moderna	AH	Ongoing symptoms despite AH (7 months)	Healthy
11 (48/F)	Negative	+	7 days	+	+	3^rd^	Pfizer‐BioNTech	AH	Ongoing symptoms despite AH (7 months)	Urticaria
12 (36/M)	Negative	+	14 days	+	+	3^rd^	Modern	AH	Ongoing symptoms despite AH (6 months)	Urticaria
13 (31/M)	Negative	Negative	14 days	Negative	+	3^rd^	Moderna	AH	Resolution after 3 months	Healthy
14 (22/F)	Negative	Negative	7 days	Negative	+	3^rd^	Moderna	AH	Lost to follow‐up	Healthy
15 (73/M)	Negative	Negative	1 day	+	+	3^rd^	Pfizer‐BioNTech	AH	Resolution 5 months	Healthy
16 (41/M)	Negative	Negative	9 days	+	+	3^rd^	Moderna	AH + CS Omalizumab	Remission, ongoing therapy AH + Omalizumab (7 months)	Lymphocytic vasculitis 4 months post vaccination

Abbreviations: AH, antihistamines; CS, systemic corticosteroids; CSU, chronic spontaneous urticaria; F, female; M, male; NSAID, non‐steroidal anti‐inflammatory drugs.

In our case series, patients from all age ranges developed delayed onset urticaria, and the reactions were more commonly observed after receiving a booster vaccination with Moderna (75%), despite only 13.5% of Danes receiving Moderna as booster vaccines.^1^ In all cases, the onset of delayed urticaria and symptomatic dermographism was temporally associated with the administration of the vaccine, although this potential adverse effect is not mentioned in the summaries of product characteristics. The mechanism is not elucidated, but the timing speaks against a type I IgE‐mediated allergic reaction. Little is known about the pathophysiological background of urticarial dermographism. We notice, that the onset of symptoms seemingly correlates with the peak responses of humoral and cellular immunity induced by vaccination. Magen et al. reported a total of 59 patients with CSU after mRNA vaccination and none of these patients had a type I IgE‐mediated hypersensitivity reaction.^2^ They found a higher frequency of allergic comorbidities and likewise almost half (43.7%) of our patients had a history of atopy. Another mechanism could be production of histamine‐releasing autoantibodies.^2^ In the preexisting literature, we could only find very few case descriptions of drug‐induced symptomatic dermographism and none of these were precipitated by vaccines. However, in recent literature we found case descriptions with delayed onset urticaria and marked dermographism after COVID mRNA‐vaccines.^5^, ^6^, ^7^ In the study by Prasad et al. reporting 14 patients with urticaria after COVID‐19 booster vaccination, 10 of these received Moderna vaccine.^4^ In a case series by Bianchi et al., 23 patients developed delayed urticarial reactions with marked dermographism and intense itch on average 9.7 days following COVID‐19 booster vaccination.^5^ The majority of their patients (91.3%) received Moderna Spikevax. The mean resolution time was 10 days, and only two of their patients developed chronic urticaria. Due to different booster dosages of Pfizer‐BioNTech (0.3 ml containing 30 μg of mRNA) and Moderna (0.25 ml containing 50 μg of mRNA), Moderna Spikevax contains more mRNA and therefore may be more immunogenic. Besides the immunogenic mRNA component, both the Comirnaty and the Spikevax vaccine contains other excipients like polyethylene glycol (PEG), which is a known cause of type I hypersensitivity as well as delayed reactions, in conjugation with lipid nanoparticles, which could potentially alter the immunogenic properties of PEG. In addition, polysorbate and tromethamine are used as excipients in the Spikevax vaccine.^8^ A reaction to PEG and polysorbate was described as a potential culprit in immediate allergic reactions initially, although the latest evidence suggests that these excipients are not relevant.^8^ Clinically it is important to identify and distinguish between immediate hypersensitivity reactions and delayed onset urticaria. The Centers for Disease Control and Prevention recommends that patients who experience immediate hypersensitivity reactions within 4 h of receiving a COVID vaccine postpone the subsequent dose until after consulting with a specialist.^3^, ^9^ The specialist should perform deep phenotyping and provide risk stratification and recommendations for subsequent vaccination based on the current available information.

In conclusion this study describes booster vaccinations with COVID mRNA‐vaccine leading to the development of a distinct clinical picture with delayed onset urticaria accompanied by marked symptomatic dermographism. These symptoms can be treated similarly to CSU, and importantly are not a contraindication to future vaccination. As many countries now are administrating a third or even fourth dose of COVID‐vaccine, we hope this letter makes clinicians aware of this potential adverse effect, especially following booster vaccination with Moderna.

## AUTHOR CONTRIBUTIONS

**Jakob Lillemoen Drivenes**: Conceptualization (Equal); Data curation (Equal); Formal analysis (Equal); Investigation (Equal); Methodology (Equal); Project administration (Equal); Supervision (Equal); Validation (Equal); Visualization (Equal); Writing – original draft (Equal); Writing – review & editing (Equal). **Aleena Banerji**: Supervision (Supporting); Validation (Supporting); Writing – review & editing (Supporting). **Anette Bygum**: Conceptualization (Equal); Data curation (Equal); Formal analysis (Equal); Investigation (Equal); Methodology (Equal); Project administration (Equal); Resources (Equal); Supervision (Equal); Validation (Equal); Visualization (Equal); Writing – original draft (Equal); Writing – review & editing (Equal).

## CONFLICT OF INTEREST

Jakob Lillemoen Drivenes has been involved in clinical studies supported by Takeda. Aleena Banerji has received a research grant from Takeda and Kalvista, and has been involved in consulting supported by Biocryst, Takeda, Kalvista, Pharvaris and Ionis. Anette Bygum has been involved in clinical studies and teaching supported by Biocryst, CSL Behring, and Takeda (former Shire).
